# Influence of the Chelation Process on the Stability of Organic Trace Mineral Supplements Used in Animal Nutrition

**DOI:** 10.3390/ani11061730

**Published:** 2021-06-10

**Authors:** Laurann Byrne, Michael J. Hynes, Cathal D. Connolly, Richard A. Murphy

**Affiliations:** 1 Alltech Bioscience Centre, Summerhill Road, Dunboyne, A86 X006 Co. Meath, Ireland; cconnolly@alltech.com (C.D.C.); rmurphy@alltech.com (R.A.M.); 2 School of Chemistry, National University of Ireland, H91 TK33 Galway, Ireland; reachforhynes@eircom.net

**Keywords:** proteinates, organic trace minerals (OTMs), enzyme hydrolysis, acidic stability, mineral absorption

## Abstract

**Simple Summary:**

Organic trace minerals (OTMs) are recognised globally as being a more bioavailable source of mineral than their inorganic counterparts. Whilst there are many forms of mineral products available for use in animal nutrition, these have unfortunately been generically entitled “organic trace minerals” by virtue of the fact that the trace elements in question are complexed or otherwise associated with organic molecules. The process of chelating transition elements such as copper, iron or zinc, for instance, typically involves reacting inorganic mineral salts with suitable bonding groups, such as a peptides or amino acids, during which the mineral becomes part of a biologically stable structure. Numerous production processes have been developed, ranging from highly specific and controlled reaction processes to more involved chemical synthesis routes. The production process used determines the product’s efficacy. Given the vastly different products that exist in the marketplace, the importance of understanding the physical and chemical differences between them cannot be overstated, and as such, manufacturing processes that affect the pH stability and functionality of the finished products were assessed in this work. The results showed that in the case of proteinate type products, the hydrolysis procedure used to generate the chelating peptides had a significant impact on proteinate pH stability, a key determinant of the product’s performance in the animal.

**Abstract:**

The effect of the chelation process on the pH-dependent stability of organic trace minerals (OTMs) used as mineral supplements in animal nutrition was assessed using analytical techniques such as potentiometry, Fourier Transform Infrared Spectroscopy (FTIRS) and amino acid profiling. The aim was to understand the influence and relative importance of the manufacturing conditions on mineral chelation and the subsequent pH stability of OTMs. A selection of OTMs were assessed over a wide pH range to account for the typical environmental changes encountered in the gastrointestinal (GI) tract. In the case of proteinate type products, the potentiometric assessment of free mineral concentration indicated that the hydrolysis procedure used to generate the chelating peptides was the major influencer of the pH stability of the products. Many products are available under the umbrella term “OTMs”, including amino acid complexes, amino acid chelates, polysaccharide complexes and proteinates. Significant differences in the pH-dependent stability of a range of commercially available OTMs were observed.

## 1. Introduction

The importance of dietary supplementation of trace minerals in animal feed is well known and various forms of both organic and inorganic products are commercially available. Organic trace minerals (OTMs) such as proteinates are known to be more bioavailable than inorganic trace mineral forms, thereby allowing more mineral to be absorbed and increasing the mineral status within the animal [1,2,3,4,5,6,7,8,9,10,11,12]. The strength of the bonds between the ligands and the mineral on the formation of a mineral chelate can prevent dissociation as it passes through the digestive system and enhance mineral bioavailability. In addition to demonstrated active uptake mechanisms [13,14], it is understood that organic trace minerals dissociate from their carrier groups in the intestinal lumen and are then absorbed into enterocytes lining the gastrointestinal tract. Reported differences in the bioavailability of organic minerals have also been attributed to the differences in dissociation rates of the mineral from the organic group to which they are bound, or to differences in mineral-chelate solubility [14].

Organic trace mineral characteristics vary between products and are determined by the type of ligands used to bond with the mineral. These ligands are usually amino acids, peptides, polysaccharides and organic acids. Although a selection of commercially available OTMs were assessed, the primary focus of this work was on proteinate products. These are produced by partially hydrolysing a protein source using either acid and/or enzymatic procedures. This results in the formation of a hydrolysate containing a mixture of amino acid and peptide ligands of varying chain length and molecular size, depending on the degree of hydrolysis. Under the appropriate conditions, the reaction of a soluble mineral salt with such a hydrolysate results in the formation of a range of mineral–ligand complexes or chelates, where the mineral is covalently bound to the ligand.

Mineral–organic ligand combinations can result in the production of one of several classes of OTMs. The Association of American Feed Control Officials (AAFCO) has classified them into groups (metal–amino acid chelates, metal–amino acid complexes, specific metal–amino acid complexes, metal proteinates and polysaccharide metal complexes), and the mineral binding properties vary greatly between them [15]. Some of these definitions and classifications have led to confusion in the industry. For example, the AAFCO defines an amino acid metal chelate as “the product resulting from the reaction of a metal ion from a soluble metal salt with amino acids, with a mole ratio of one mole of metal to one to three (preferably two) moles of amino acids to form coordinate covalent bonds”. Since this definition does not consider the actual binding in the complex, it is possible that a complex considered a chelate by animal nutritionists might not be considered as such by a chemist.

In the case of mineral proteinates, which are the subject of this work, the efficiency of the hydrolysis procedure and the choice of enzyme are only two of many factors that can affect the stability of the final product. An effective hydrolysis procedure is essential to mineral–proteinate production as it cleaves the peptide bonds in the protein source, thereby providing soluble amino acids and small peptides to which the mineral ion can bind. The extent of hydrolysis varies depending on the enzyme used and hydrolysis conditions such as pH, temperature, time, the enzyme-to-substrate ratio and the solid-to-liquid ratio.

## 2. Materials and Methods

All materials used were of ACS grade or higher where appropriate. Chemicals were supplied by Sigma-Aldrich, Tallaght, Ireland. The soy flour protein source was provided by Alltech Bioscience Centre, Dunboyne, Co. Meath, Ireland. Enzymes and commercial OTMs were sourced from independent distributors.

### 2.1. Protein Source Hydrolysis

Protein hydrolysates were prepared in the laboratory using a range of commercially available enzymes as described previously [16]. For acidic hydrolyses, the pH was maintained at pH 3.0 ± 0.2 and the temperature at 55 ± 1 °C. Alkaline hydrolyses were carried out at pH 8.5 ± 0.2 and 55 ± 1 °C. pH adjustments were carried out using sodium hydroxide (4M NaOH) or hydrochloric acid (conc. HCl) to increase or decrease pH, respectively. Combinations of the selected enzymes were also tested to determine if the hydrolysis procedure was improved with the additional mixtures of ligands produced from the different enzyme’s mode of action.

### 2.2. Formation of Mineral Proteinates from Soy Flour Hydrolysate

The sulphate form of the element was used to prepare batches of mineral proteinates with a final mineral content of 10% (*w*/*w*) when dried. Copper was selected as the element for producing the proteinates as it was possible to measure the free mineral concentration in solution directly by potentiometry. Mineral salt was added to soy hydrolysate (90 mL) and the contents were mixed for 1 h at room temperature. The pH of each proteinate was recorded and the samples were then cooled and frozen at −70 °C prior to lyophilisation. The dried powders were homogenised using a pestle and mortar and stored in sterile, high density polyethylene (HDPE) airtight containers. The mineral content of the dried product was confirmed using Inductively Coupled Plasma Optical Emission Spectroscopy (ICP-OES) [17]. Commercial samples of copper (II) proteinates were analysed as received.

### 2.3. Amino Acid Profiling

A free amino acid analysis was carried out at Alta Bioscience, Birmingham, UK, by cation chromatography on a Biotronic LC 5001 automated amino acid analyser, according to a modified method (24 h at 110 °C) of Spackman et al. [18].

### 2.4. Fourier Transform Infrared (FTIR) Spectroscopy

Fourier Transform Infrared Spectroscopy (FTIRS) spectra were recorded on a PerkinElmer Spectrum 100 FT-IR spectrometer equipped with a Universal Attenuated Total Reflectance (UATR) sampling accessory. All samples were ground using an agate mortar and pestle prior to loading to ensure homogeneity. Samples were pressed onto the UATR crystal at a force of 80 N. Spectra were recorded in the range 1800–650 cm^−1^ with a resolution of 4 cm^−1^ and an average of 16 scans/sample was recorded. To avoid cross-contamination, the accessory was washed with deionised water and wiped dry with a non-static laboratory wipe between samples. An air background was collected prior to each analytical run. Analysis was carried out at room temperature. The FTIR spectra were subjected to ATR and automatic baseline correction before further analysis.

### 2.5. Potentiometric and Ion-Selective Electrode (ISE) Measurements

Potentiometric titrations were carried out using a combination of two Orion Star Series pH-meters with a precision of 0.1 mV or 0.001 units of pH. One of the meters monitored pH using a combination glass electrode and an automatic temperature controller. The second meter measured the electromotive force (EMF) and was connected to a Jenway copper combination ion selective electrode (ISE) with a solid-state crystalline membrane and an integral driTEK reference electrode (Barloworld Scientific Ltd., Stone, UK). Standard calibrations were carried out immediately prior to experimental work. Lyophilised samples (0.25 g) were prepared for analysis by resuspension in 25 mL distilled water. Titrations were performed in a 50 mL double-walled glass vessel. The temperature inside the cell was maintained at 25 ± 0.1 °C by water circulation through the vessel. Sodium nitrate (0.1 M NaNO_3_) was used to maintain a constant ionic strength. Titrations were carried out in triplicate. Measurements above pH 8 were not taken due to mineral solubility concerns as well as it being outside of the recommended working range (pH 3–8) of the ISE.

### 2.6. Statistical Analysis

Data were analysed by statistical means using the programs Minitab version 20 (Minitab Ltd., Coventry, UK) and The Unscrambler version 10.5 (Camo Analytics, Oslo, Norway). A one-way analysis of means (ANOM) was used to assess differences between means.

## 3. Results and Discussion

The benefits of incorporating OTMs in feed are widely accepted, but the effectiveness of such products relies on preserving the chemical form of the mineral until it can be absorbed into the bloodstream. A large proportion of bioactive compounds are poorly available due to factors such as low permeability and/or solubility within the gut, lack of stability during feed processing (temperature, oxygen, light) and challenges in the gastrointestinal tract such as pH conditions, the presence of enzymes or unwanted interactions with other nutrients [19,20,21,22].

The aim of this work was to determine the effect of protein hydrolysis conditions on the pH-stability of trace mineral proteinates. Enzymes cleave peptide bonds specifically, depending on their modes of action and on their amino acid recognition sequences. Some enzymes are more efficient with respect to generating suitable peptide fragments for chelation with a mineral ion [23,24,25]. In this work, the soy protein was hydrolysed using a variety of enzymes and enzyme combinations (Table 1). Alkaline and acidic enzymes outlined in Table 1 were investigated individually and in combination. Sequential hydrolyses (Samples 11 and 12, Table 1) were also tested, giving each type of enzyme an opportunity to work separately under optimum conditions as determined previously [16]. Several studies have shown that varying the enzyme type during hydrolysis results in mixtures of ligands with different chemical properties and abilities to chelate minerals [24,25,26,27,28,29,30,31].

**Figure 1:**
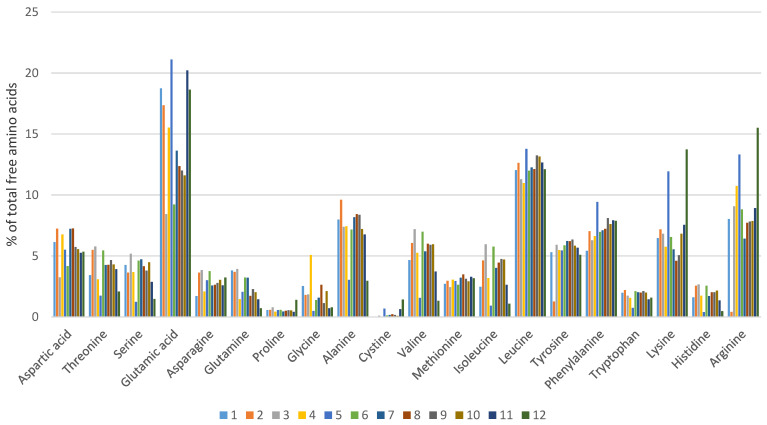
Free amino acid profiles of soy protein following enzymatic hydrolysis under different conditions. 1–12 refer to the individual hydrolysates outlined in Table 1.

The free amino acid profile of each hydrolysate is shown in Figure 1. There were some differences in the concentrations of individual amino acids across the 12 hydrolysis conditions. However, the overall patterns were consistent, probably due to the raw material being the same in each case. It was not possible to attribute the subtle differences in the free amino acid profile to the hydrolysis conditions so amino acids were grouped into categories based on their side chains (R-group) for subsequent statistical analysis. The groups chosen were acidic (A), basic (B), polar (P), neutral (N) and combinations thereof. Again, no significant differences (*p* > 0.05) were observed with regard to the amino acid categories under different hydrolysis conditions (Table 2). These results indicated that amino acid profiling alone does not allow for differentiation between mineral proteinates prepared from enzyme hydrolysates of soy flour.

**Figure 2:**
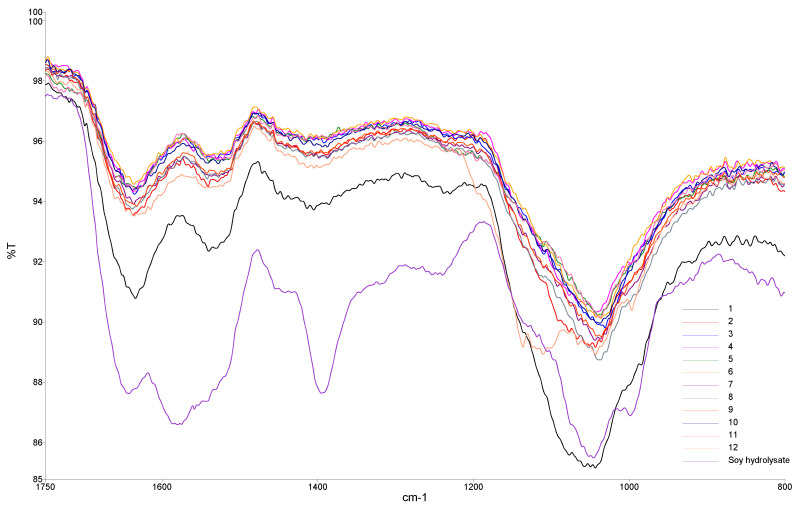
FTIR spectral overlays of copper proteinates formed from a variety of enzymatic hydrolysis procedures. 1–12 refer to the individual hydrolysates outlined in Table 1.

FTIR spectroscopy was used to discriminate between the different products formed from the mineral proteinates created from the 12 individual soy hydrolysates (Figure 2). Differences were observed in the fingerprint region of these spectra, indicating that the hydrolysis procedure had an effect on the presence of certain functional groups, which, in turn, could have an impact on the ligand mixture’s ability to interact with the transition metal ion. For example, carboxylic oxygen and amino nitrogen atoms are involved in the formation of mineral–ligand bonds, so band shifts in these regions should accompany such interactions [32]. These observations agree with previous work on the quantitative analysis of the bound mineral in copper proteinates using ATR-FTIR and powder X-ray diffraction (PXRD) [33].

**Figure 3:**
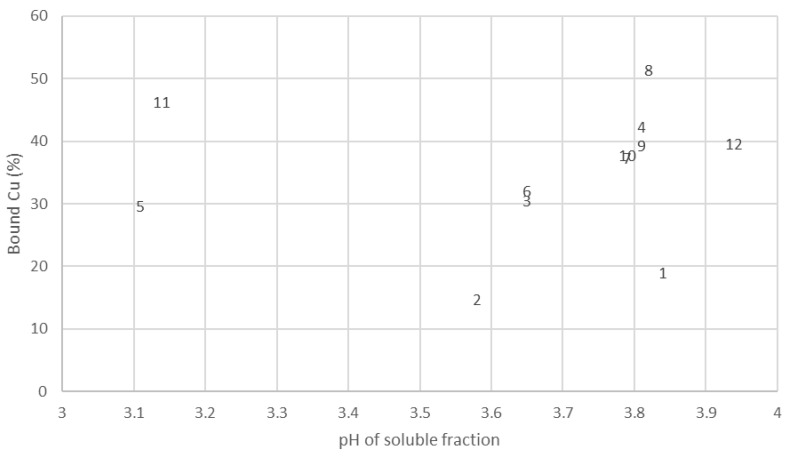
Scatter plot of initial pH and corresponding bound Cu (%) for mineral proteinates. 1–12 refer to the individual hydrolysates outlined in Table 1.

**Figure 4:**
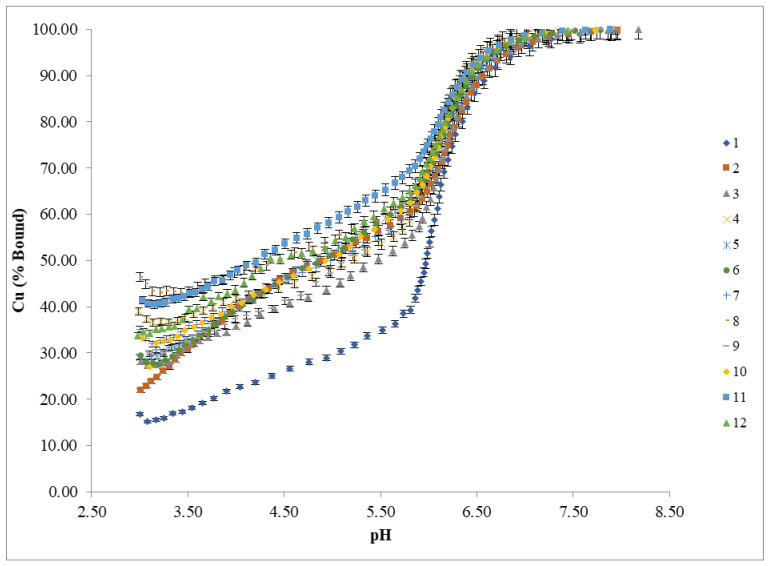
ISE titrations (*n* = 3) of bound Cu in proteinates containing 10% (*w/w*) Cu. 1–12 refer to the individual hydrolysates outlined in Table 1.

A potentiometric analysis was used to measure the concentration of free (unbound) copper in aqueous preparations of the test samples. The scatter plot in Figure 3 shows the initial bound copper in the laboratory-manufactured proteinates relative to the product pH. The results suggest that there are pH-independent differences in mineral-binding abilities of the hydrolysates. Bound copper concentrations in the test proteinates varied between a low of 14.75% (Hydrolysis 2) and a high of 51.41% (Hydrolysis 8). Hydrolysates from multiple enzymes had higher bound copper levels (Figure 3), a trend that was observed when the pH of these proteinates was lowered to pH 3 for the potentiometric titration (Figure 4).

Due to the differences in the pH of each of the test proteinate soluble fractions, each was adjusted to pH 3 prior to potentiometric titration in order to standardise the starting point while staying within the operating range of the ion selective electrode. During the titration, aliquots of base (0.1 M NaOH) were added incrementally to allow for the measurement of free Cu^2+^ concentrations up to pH 8. This allowed for the bound copper (%) to be calculated across the pH range from 3 to 8 (Figure 4).

It is apparent from the data in Figure 4 that there are distinct variations in the stability of the various laboratory-manufactured proteinates across the pH range studied. Hydrolysates formed through the use of multiple enzymes appear to have the highest mineral binding ability as there is less free Cu^2+^ detected by the ISE over the course of the titration. This correlates with the scatter plot results of initial pH values and percentage mineral bound in the 12 proteinates (Figure 3).

**Figure 5:**
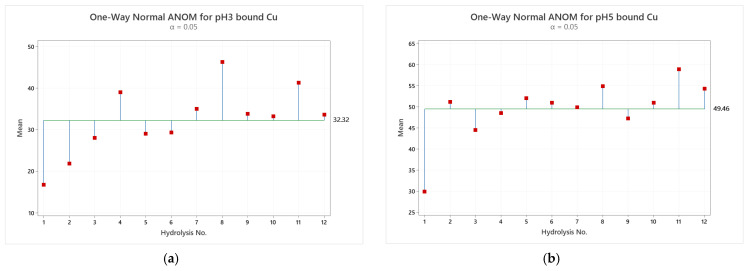
(**a**) One-Way Analysis of Means for bound Cu at pH 3; (**b**) One-Way Analysis of Means for bound Cu at pH 5. 1–12 refer to the individual hydrolysates outlined in Table 1.

Based on the results of the ISE titrations, the free amino acid profiling results obtained for each enzymatic hydrolysis (Figure 1) were further assessed to determine if any correlation existed between the free amino acid groups and the percentage of bound Cu in the samples. This enabled assessment on whether the mineral was associated with free amino acids produced from the various enzymatic hydrolyses. The classes of amino acids, and the combinations of classes of amino acids were assessed by a One-way Analysis of Means. Figure 5a,b illustrate the One-way Analysis of Means at pH 3 and pH 5 of the different hydrolysates in relation to the level of bound Cu. The secondary dataset (pH 5) was chosen to reflect the average pH in the duodenum, which is the main site of absorption of trace minerals. Overall, this data shows no clear correlation between the level of bound copper and/or grouped amino acids. These results reinforce the proposition that mineral binding is not significantly dependent on the level and extent of free amino acids, but it is other protein-derived hydrolysis products (peptides) that play a central role with respect to mineral chelation in proteinates.

**Figure 6:**
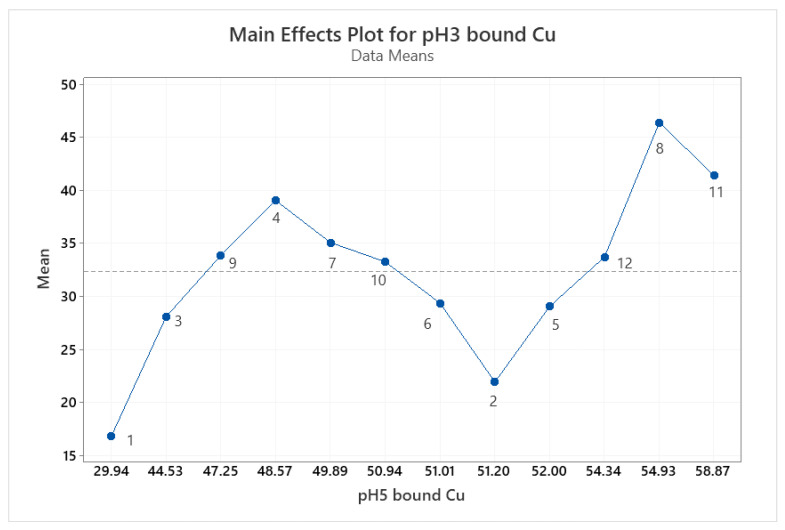
Main effects plot for bound Cu at pH 3 vs. pH 5. The values 1–12 represent the average value obtained from results obtained at pH 3 and pH 5 for the 12 individual hydrolysates in Table 1.

Figure 6 shows a main effects plot for bound mineral at pH 3 and pH 5. Results are plotted from low to high in terms of bound Cu results at each pH. As the pH is increased from 3 to 5, there is a general trend towards more bound copper present, however, this does not hold true in all cases, indicating the impact of the hydrolysis procedure itself has a greater effect on the bound mineral. Hydrolysis 8, which combines both acidic and alkaline enzymes, had the highest bound copper at both pH values. Reviewing the percentage of each individual amino acid present in this hydrolysis compared to the other eleven (Figure 1), none were present in significantly higher or lower levels that might account for the observed increase in mineral binding. Similarly, no clear benefit was noted with the use of acidic or alkaline enzymes in terms of enhancing mineral binding and no correlation between the extent of mineral binding and the use of multi-enzyme hydrolysis procedures was observed.

Certain individual amino acids can have greater affinity for Cu ions under specific conditions and can contribute to mineral binding [34,35]. In this study, the differential ability of proteinates to bind mineral stably appeared not to be dependent on the presence of amino acids either individually or based on their R-group. The enzymatic hydrolysis of protein will result in the release of free amino acids along with short chain peptides, both of which can act as mineral binding ligands. The amino acid configuration in a peptide can significantly influence their ability to interact with trace minerals and consequently affect the overall stability of resultant chelates [25,36,37,38,39,40,41]. To illustrate this point, Table 3 highlights the stability constants for a number of tripeptides complexed with Cu(II) under identical physiological conditions. Logβ refers to the log of the stability constant and the corresponding subscript refers to the metal:ligand ratio.

Table 3 shows that the sequence and position of amino acids within a peptide can have a significant impact on stability. The substitution of histidine into the tripeptide Gly–Gly–Gly to yield Gly–Gly–His, for instance, enhances the stability and thus the relative proportion of bound copper. Changing the position of this histidine within the tripeptide sequence (to form Gly–His–Gly, for example) can result in a further increase in the bond strength and, as such, an increase in the proportion of the bound mineral. In practical terms, simple changes in the configuration of amino acids in this tripeptide result in a greater proportion of bound mineral relative to free mineral and ligands. Previous work assessed the effect of the positional substitution of histidine in the tripeptide sequence, in addition to longer chain peptide sequences, and factors such as folding and structural rearrangements (e.g., the formation of fused rings) can also contribute to enhanced stability [16,43]. Essentially, proteinate stability can be significantly influenced not only by the type of peptide used, but also the configuration of amino acids in a peptide sequence.

To generate proteinates with effective mineral binding capabilities, the choice of enzyme is important with respect to obtaining a hydrolysate containing peptides with strong potential as ligands for chelation. All enzymatic hydrolysates chosen for this work were capable of binding mineral ions, however little correlation was observed between ability to bind copper and the free amino acid profiles of the individual proteinates. Several studies have shown that strongly coordinating side chain residues in appropriate locations can behave as anchors for the coordination of the amide donors of peptides [24,25,44,45,46,47] and will therefore play a much greater role in influencing the ability of proteinates to stably bind mineral.

### Potentiometric Titrations of Commercial OTMs

**Figure 7:**
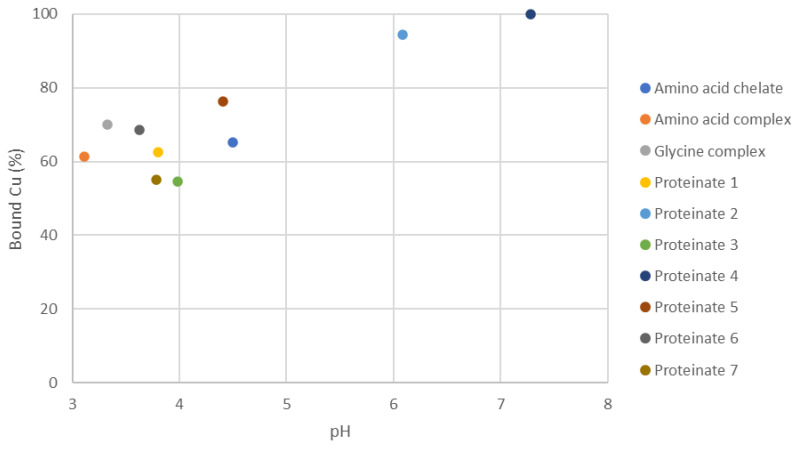
Scatter plot of initial pH of commercial OTMs and their respective bound copper values.

Commercially available organic trace mineral products were assessed to determine whether or not potentiometric titrations could assist with product differentiation based on their different manufacturing processes. A range of products containing 10.34 to 19.09% (*w*/*w*) Cu were analysed. Aqueous suspensions of these products had highly variable pH values, thus reflecting the different procedures used during their manufacture (Figure 7).

**Figure 8:**
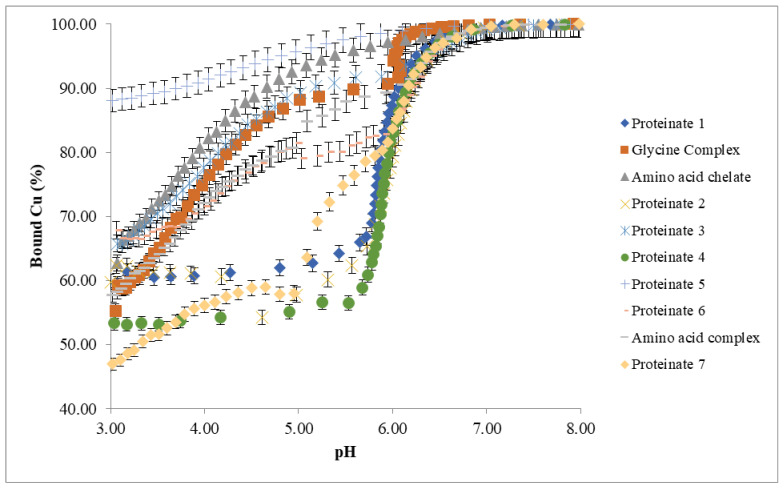
ISE titrations (*n* = 3) of commercial copper OTMs.

Most of the samples were seen to form an insoluble precipitate above pH 5.5, and this phenomenon can be observed as inflections in the curves for these OTMs in Figure 8. When this occurred, it was not possible to continue with the titration.

The commercial samples tested during this study were manufactured using different procedures. Details of these processes were not known by the researchers, other than that the proteinate products were manufactured from hydrolysed plant protein and some form of copper salt. These differences were observed as variability in bound copper concentrations. Previous work assessing a selection of commercially available organic copper products also showed significant differences with bound Cu, varying between 16 and 94% [48].

## 4. Conclusions

Production processes vary greatly between manufacturers of organic trace minerals and these differences result in products with a wide range of properties, even though they may fall under the same classification and use common terminology. Such products are not expected to be equally effective from a nutritional perspective and the rates of mineral uptake and retention by the animal are likely to differ greatly between them. This work focused on differentiation between mineral (copper) proteinates by directly measuring their ability to bind and stabilise the mineral under pH conditions that may be encountered during digestion. In the case of proteinates that are produced from enzymatically hydrolysed plant protein, it was found that changes to the hydrolysis conditions resulted in products with different concentrations of bound mineral. At first, these changes were thought to be attributable to the free amino acid profile of the hydrolysed protein. However, further investigations showed this not to be the case, and instead, it is the mixture of ligands, which includes not only amino acids but also peptides and soluble polypeptides, that accounts for much of the differences in mineral-binding ability between the products. The analysis of commercially available mineral proteinates allowed for discrimination between them based on their bound mineral concentrations and their responses to pH changes during potentiometric titrations.

## Figures and Tables

**Table 1:** Enzymes selected for protein hydrolyses.

Hydrolysate Number	Enzyme Used ^1^
1	Cysteine Protease 1
2	Serine Protease
3	Fungal Protease
4	Cysteine Protease 2
5	Acidic Fungal Protease
6	Serine Protease + Fungal Protease
7	Serine Protease + Cysteine Protease 1
8	Serine Protease + Cysteine Protease 2
9	Serine Protease + Acidic Fungal Protease
10	1–5–Multi enzyme combination
11	2 + 5–Alkaline followed by acidic hydrolysis
12	5 + 2–Acidic followed by alkaline hydrolysis

^1^ All enzymes were sourced from independent distributors.

**Table 2:** Descriptive statistics for amino acid group data and different hydrolysis procedures using Minitab.

Variable	AA Group ^1^	N	Mean	SE Mean	StDev	Minimum	Q1	Median	Q3	Maximum
%	A + N	12	64.45	1.82	6.29	56.28	59.27	65.07	66.33	78.28
	A + P	12	39.488	0.748	2.590	36.452	37.163	38.470	42.278	43.411
	Acidic	12	20.70	1.41	4.87	11.68	17.32	21.57	24.82	26.62
	B + A	12	38.53	2.27	7.85	30.23	33.00	34.65	42.74	53.71
	B + N	12	61.593	0.973	3.370	57.419	58.216	61.945	63.406	69.486
	B + P	12	36.63	1.32	4.57	27.90	34.19	34.97	40.72	43.77
	Basic	12	17.84	1.52	5.26	10.15	14.50	17.36	18.48	29.72
	Neutral	12	43.76	1.73	5.99	32.29	40.59	45.28	47.21	53.39
	P + N	12	62.55	2.41	8.35	46.34	57.39	65.68	68.34	71.91
	Polar	12	18.792	0.950	3.293	14.051	16.133	18.876	20.890	24.775

^1^ For the amino acid (AA) groupings outlined, A = Acidic, B = Basic, N = Neutral and P = Polar.

**Table 3:** ML ^1^ stability constant values for a selection of tripeptides ^3^.

Tripeptides	logβ_ML_^1,2^
Gly-Gly-Gly	5.13
Gly-Gly-His	7.55
Gly-His-Gly	9.25
Gly-His-Lys	16.44
Gly-Gly-Tyr	10.01

^1^ ML refers to the Metal:Ligand ratio; ^2^ logβ refers to the log of the stability constant and the corresponding subscript refers to the Metal:Ligand ratio. ^3^ Values sourced from National Institute of Standards and Technology database [42].

## Data Availability

Data is contained within the article.
